# Demographic analysis of continuous-time life-history models

**DOI:** 10.1111/j.1461-0248.2007.01121.x

**Published:** 2008-01

**Authors:** André M De Roos

**Affiliations:** Institute for Biodiversity and Ecosystem Dynamics, University of Amsterdam PO Box 94062, NL-1090 GB Amsterdam, The Netherlands

**Keywords:** Continuous time, dynamic energy budget model, life history, Lotka's integral equation, population growth rate, sensitivity

## Abstract

I present a computational approach to calculate the population growth rate, its sensitivity to life-history parameters and associated statistics like the stable population distribution and the reproductive value for exponentially growing populations, in which individual life history is described as a continuous development through time. The method is generally applicable to analyse population growth and performance for a wide range of individual life-history models, including cases in which the population consists of different types of individuals or in which the environment is fluctuating periodically. It complements comparable methods developed for discrete-time dynamics modelled with matrix or integral projection models. The basic idea behind the method is to use Lotka's integral equation for the population growth rate and compute the integral occurring in that equation by integrating an ordinary differential equation, analogous to recently derived methods to compute steady-states of physiologically structured population models. I illustrate application of the method using a number of published life-history models.

## Introduction

Computation of population growth rate and its sensitivity to changes in vital rates (e.g. survival, growth, development and reproduction) is an important approach in conservation biology (e.g. Morris & Doak 2002), ecotoxicology (e.g. Kooijman & Metz 1984; Klok *et al.* 1997), pest management (e.g. Shea & Kelly 1998), as well as evolutionary ecology (e.g. van Tienderen 2000). These analyses most often exploit matrix models (Caswell 2001) to project long-term changes in population density under the assumption that environmental conditions and hence vital rates remain unchanged. Matrix models allow for an easy computation of the asymptotic population growth factor lgr as the dominant eigenvalue of the projection matrix [here and below I will reserve the term *population growth rate* for the quantity *r* = log (lgr), as technically lgr itself is not a rate]. lgr succinctly summarizes the influence of life-history components on population performance. The dominant right and left eigenvector of the matrix represent the stable distribution of the exponentially growing population and the reproductive value of individuals in different life-history stages, respectively (Caswell 2001). The eigenvectors can moreover be used to compute the sensitivity of lgr to changes in vital rates (Caswell 1978). Expressions for these measures of population performance (growth factor lgr and its sensitivity, stable distribution and reproductive value) were first derived for constant environments and later extended to environments in which vital rates vary periodically (Caswell & Trevisan 1994) or stochastically (Tuljapurkar 1990) or are influenced by population density (Takada & Nakajima 1992).

Matrix models are based on two types of discretization: (i) the population is subdivided into distinct classes or stages, most often age, size or stage of development and (ii) population dynamics are described on a discrete-time basis. Subdivision into population stages inevitably introduces discretization errors if individuals are in reality classified by a continuously varying trait such as body size. Methods have been developed to objectively select a stage classification that most faithfully represents such a continuous population distribution (Vandermeer 1978), but these approaches at best minimize the problems that these errors introduce into the population projection and do not eliminate them (Easterling *et al.* 2000; Pfister & Stevens 2003). Integral projection models (Easterling *et al.* 2000; Ellner & Rees 2006) are akin to matrix models, but represent the population by a continuous distribution and thus solve the problems associated with discrete stages. Ellner & Rees (2006) summarize basic theory and computational details for integral projection models, analogous to the theory for matrix population models (Caswell 2001), in which individuals are classified by an arbitrary number of variables.

Both matrix and integral projection models describe dynamics on a discrete-time basis, projecting the population state from time *t* to some future time *t* + 1. This time discretization has at least two advantages. Observational data on survival, reproductive output and stage transitions of individuals are usually collected on a discrete-time basis as well and can hence be used directly to parameterize the models. In addition, by parameterizing the population model on the basis of such observational data it also accounts phenomenologically for any unexplained and possibly stochastic variability in vital rates among individuals in the same stage. Matrix and integral projection models are therefore the ideal tool to extrapolate observations on vital rates to future population performance. However, demographic analyses may also start out by formulating a mathematical model of the individual life history, such as a dynamic energy budget (DEB) model (Kooijman & Metz 1984; Kooijman 2000), and address the question how particular assumptions about life history affect population performance (e.g. Kooijman & Metz 1984; Klok & De Roos 1996; Hulsmann *et al.* 2005; Rinke & Vijverberg 2005). Formulating a matrix population model may then be complicated, if the individual-level dynamics are described on a continuous-time basis. For example, Klanjscek *et al.* (2006) construct a projection matrix on the basis of a DEB model. To compute the matrix entries the stable age distribution within a particular life-history stage has to be evaluated, which itself depends on the population growth factor lgr (see also Caswell 2001, p. 164). As a consequence, the matrix construction and computation of lgr requires a rather complex, multistep iterative process (see also Klok & De Roos 1996; Klok *et al.* 1997, for an earlier example).

If the model of individual life history treats time as a continuous variable, physiologically structured population models (Metz & Diekmann 1986; De Roos 1997) are a more natural choice to describe demography. Physiologically structured population models describe the changes of a continuous population distribution on a continuous-time basis using either partial differential equations (Metz & Diekmann 1986; De Roos 1997) or integral equations (Diekmann *et al.* 1994). However, to compute the population growth rate for such a model requires solving an integral equation, which in its simplest form can be written as: (1)
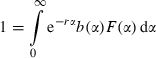


This is Lotka's integral equation for calculating the population growth rate *r* [= log (lgr)], in which *b*(*a*) represents the individual fecundity at age *a* and *F*(*a*) the survival probability up to that age. Practical applications of this equation have commonly involved a discretization of the integral occurring in its right-hand side (e.g. Michod & Anderson 1980; Taylor & Carley 1988; Stearns 1992). Caswell (2001, p. 197) discourages this approach and argues that formulating a discrete-time, matrix model should be preferred over any attempt to write discrete versions of Lotka's equation. A condition similar to Lotka's integral equation determines the steady-state of a structured population model in case the population would not grow exponentially but would approach equilibrium as a result of some form of density dependence (see Metz & Diekmann 1986; De Roos *et al.* 1990; De Roos 1997, for examples). For such steady-state analysis of physiologically structured populations Diekmann *et al.* (2003, see also Kirkilionis *et al.* 2001) recently derived methods, in which the integral in the steady-state condition is computed without discretizing it (see Claessen & De Roos 2003 for a more intuitive explanation). In this paper, I exploit the similarity between the aforementioned steady-state condition and Lotka's integral equation to adapt these methods and present a simple computational approach to compute the population growth rate *r* and its associated statistics (sensitivities, stable distribution and reproductive value) using Lotka's integral eqn 1 or generalized versions of it. The idea behind the approach is to evaluate the integral in the right-hand side of the equation by means of numerical integration of an ordinary differential equation (ODE). The method is generally applicable to analyse population growth and performance for a wide range of individual life-history models, provided individual development throughout life is deterministic and the population is growing exponentially in a constant or periodically varying environment. I will illustrate the method using published life-history models and show how it can be applied to cases of varying complexity, including situations in which individuals vary in their trait values and situations in which the life history is characterized by discrete reproduction but development is continuous in time.

## Computing the population growth rate

### The basic idea

Given that no single individual lives forever, Lotka's integral equation for the population growth rate *r* is more appropriately specified as: (2)
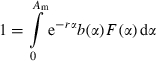


In this equation *A*_m_ represents the maximum age an individual can possibly attain or at which the survival function has become indistinguishable from 0: *F*(*A*_m_) ≈ 0. In practice, if the survival function only becomes equal to 0 in the limit *a*→∞, one has to choose a threshold age beyond which further contributions to the integral in eqn 2 become negligible. In all examples presented below I have used as a maximum age the value *A*_m_ that satisfies the condition *F*(*A*_m_)e^−*rA*_m_^ = 1.0×10^−9^.

Define the function *L*(*a*,*r*) as follows: (3)
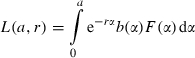


*L*(*a*,*r*) resembles the integral in Lotka's equation but up to age *a*, as opposed to the maximum age *A*_m_. Here and below, I consistently include *r* as a function argument of *L*(*a*,*r*) to indicate that *r* is the variable of interest we want to solve for. The simple idea underlying the method I present is to compute *L*(*a*,*r*) by numerical integration of the following ODE, which is derived by differentiating the expression for *L*(*a*,*r*) with respect to *a*: (4)
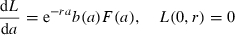


Provided explicit expressions for the birth rate *b*(*a*) at age *a* and the survival probability *F*(*a*) up to that age and given a value for *r*, this ODE can readily be solved using any kind of mathematical software packages (matlab, maple) up to the maximum age *A*_m_. The population growth rate is now that particular value of *r*, which satisfies: (5)
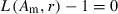


This last equation can be solved using a root-finding method, such as the secant or Newton-Raphson method (Press *et al.* 1988) that are standard parts of all mathematical software packages (matlab, maple). Because *L*(*A*_m_,*r*) is a monotonously decreasing function of *r*, these methods moreover readily converge to the required root, which I will indicate with 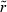. Solving eqn 5 is like finding the root of any other type of nonlinear equation in one unknown variable, except that whenever the function *L*(*A*_m_,*r*) in the left-hand side of the equation has to be evaluated we have to integrate ODE (eqn 4) numerically with the current estimate of *r*.

Once the population growth rate 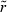
has been found, it is relatively easy to also compute all other quantities that are of interest in a demographic analysis – stable age distribution, reproductive value and sensitivity and elasticity of the population growth rate with respect to parameters. The stable age distribution is given by the explicit expression: (6)
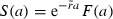
in which *S*(*a*) represents the density of individuals with age *a* in the exponentially growing population relative to the density of newborn individuals. Similarly, the reproductive value *v*(*a*) – i.e. the average contribution of an individual of age *a* to future generations relative to its contribution as a newborn – is given by (Fisher 1930, p. 27 and Metz & Diekmann 1986, pp. 155–156): (7)
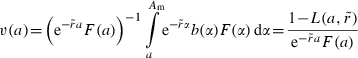


Obviously, to compute *v*(*a*) for a particular age we have to calculate 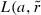) and hence we need to integrate the ODE in eqn 4 numerically up to that particular age using the computed value of the population growth rate 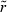. Finally, an expression for the sensitivity of the population growth rate 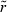
to small changes in a particular parameter *p* can be derived by differentiating both sides of eqn 5 with respect to *p*, while taking into account that the population growth rate 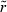
depends on *p* as well: 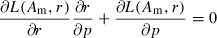


As long as the population growth rate is uniquely determined, which it generically is, the derivative ∂*L*/∂*r* is unequal to 0. The sensitivity of the population growth rate 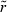
with respect to *p* can therefore be computed as: (8)
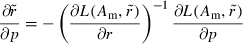


As analytical expressions for the derivatives of the function *L*(*A*_m_,*r*) are lacking, the differentials in eqn 8 have to be calculated numerically. For example, to obtain ∂*L*/∂*r* we would compute: 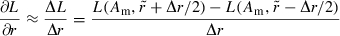


This computation involves two additional integrations of the ODE in eqn 4 with the slightly perturbed values 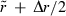
and 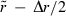, in which Δ*r* is a small step size (e.g. 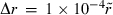). Sensitivities can subsequently be translated into elasticities using the standard definition 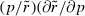). All these computational steps are readily implemented in software packages like matlab or maple. Generic matlab scripts for this purpose are provided as Supplementary Material to this paper.

The above presentation of the method focuses on the issue of computing the integral in Lotka's integral equation by means of numerical integration. However, the real power of the method lies in its applicability to situations without explicit functions for the individual fecundity at age *a*, *b*(*a*) and the survival probability up to that age *F*(*a*), respectively. For example, if fecundity and survival are the outcome of a more complicated model of individual life history, such as a DEB model, or if newborn individuals exhibit some variation in their physiological traits. In the following examples I will show how the method covers these more complex situations.

### A simple age-dependent example

This example illustrates the simplest extension of the basic idea presented above, in which mortality of individuals is specified by a daily mortality rate *m*(*a*). As case study I use the life history of the Mediterranean fruitfly (*Ceratitis capitata*) or Medfly in short, which has been extensively studied and documented (e.g. Carey 1993; Müller *et al.* 2001). Carey (1993, p. 159) shows that the daily mortality rate of medflies increases exponentially with age and provides parameter estimates for this relationship between mortality rate and age. Müller *et al.* (2001) present fecundity data, which are best described by an exponentially declining fecundity as a function of the time since the onset of reproduction. Table 1 specifies the life-history equations and parameter estimates as derived by Carey (1993) and Müller *et al.* (2001).

**Table 1:** Model equations, parameters with default value and interpretation, and population growth rate results for the Medfly example model (Carey 1993; Müller *et al.* 2001)

Life-history equations		
Fecundity:	*b*(*a*) = bgr _0_e^− bgr _1_(*a*−*A*_*j*_)^	if *a* > *A*_*j*_ (19)
Mortality rate:	*m*(*a*) = mgr _0_e^mgr_1_*a*^	(20)
**Parameter**	**Value**	**Description**
β _0_	47.0 day^−1^	Maximum daily fecundity right after maturation
β _1_	0.04 day^−1^	Decay rate in fecundity with age
*A*_*j*_	11.0 day	Age at first reproduction
μ _0_	0.00095 day^−1^	Daily mortality rate of newborn individuals
μ _1_	0.0581 day^−1^	Rate of increase of mortality with age
**Results**		
Population growth rate:	0.41906	(+4.0×10^−5^%)
Sensitivity to bgr _0_:	0.0016159	(−9.3×10^−4^%)
Sensitivity to bgr _1_:	−0.16460	(+6.3×10^−3^%)
Sensitivity to *A*_*j*_:	−0.031982	(+7.6×10^−4^%)
Sensitivity to mgr _0_:	−1.5264	(+9.1×10^−4^%)
Sensitivity to mgr _1_:	−0.011325	(+7.6×10^−4^%)

Percentages in parentheses represent the difference between the result computed with the integration method and the analytical values derived in Appendix 1.

The survival probability *F*(*a*) up to age *a* is related to the instantaneous mortality rate *m*(*a*) by the following relationship (Metz & Diekmann 1986; De Roos 1997): 
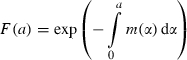


Differentiating this relationship with respect to *a* reveals that *F*(*a*) is the solution of the ODE d*F*/d*a* = −*m*(*a*)*F* with initial condition *F*(0) = 1. Lacking an explicit expression for *F*(*a*) its value can hence be computed by integrating the latter ODE at the same time as integrating the ODE for *L*(*a*,*r*). In practice, it is often numerically more efficient to solve an ODE for the stable age distribution *S*(*a*) (eqn 6) instead of the survival probability *F*(*a*) as it saves multiplication by the factor exp(−*ra*) and in addition yields the stable age distribution as immediate result of the integration. Instead of solving the ODE in eqn 4 with an explicit expression for the survival probability *F*(*a*) the value of *L*(*A*_m_,*r*) in eqn 5 can therefore also be computed by solving the following system of two differential equations: (9)
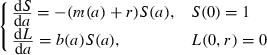
 The matlab code in Appendix S4 uses this approach to calculate the population growth rate and the sensitivities of the population growth rate with respect to the different life-history parameters, while Table 1 summarizes the results of these computations.

The exponentially increasing mortality rate of medflies with age allows for deriving an explicit expression for *F*(*a*), yielding a Gompertz survival function (Carey 1993, p. 159). As shown in Appendix 1 this in turn allows for the derivation of a nonlinear, but analytical expression of Lotka's equation. The latter I used as an alternative method to compute the population growth rate and its sensitivities to the five model parameters (see Appendix 1). In addition to the population growth rate and the parameter sensitivities obtained by integration of the ODEs in eqn 9 and solving eqn 5, Table 1 also presents the deviations of these results from the analytical values obtained by means of the approach presented in Appendix 1. The estimates for the population growth rate obtained with the two different methods differ < 0.0001% from each other, while the relative differences in parameter sensitivities range from 0.0007% to 0.006%. The results of the computational approach using integration obviously compare very well with the analytical results given that the discrepancies are of the same order of magnitude as the relative accuracy (0.0001%) with which the numerical solution of the ODEs in eqn 9 is computed.

As yet another approach the population growth rate can also be estimated as the dominant eigenvalue of an age-classified matrix model, in which the stage transition and fertility entries are estimated from the individual survival function *F*(*a*) and fecundity function *b*(*a*) (see Appendix 1). As we are dealing with continuous reproduction and hence a continuous population age distribution deriving these matrix entries requires an assumption about the projection interval, which at the same time determines the age classification. I used the relationships presented by Caswell (2001, eqns 2.24 and 2.34 on pp. 23–25) to compute these matrix entries, assuming a maximum age of 50 days, which approximately equals the maximum age in the integration of the ODEs in eqn 9. With a projection and age-classification interval of 0.5, 1.0, 2.0 and 4.0 days the population growth rate predicted by the matrix model differed 12.4%, −0.13%, −4.4% and 2.43%, respectively, from the analytically derived value (see Appendix 1). The matrix model hence yields an estimate of the population growth rate that deviates much more from the analytical value than the estimate obtained through the integration approach presented in this paper. In addition, the deviation depends on the choice of the projection and age-classification interval in such a way that a smaller projection interval does not necessarily yield a better estimate. For populations with continuous reproduction the integration approach thus yields more reliable estimates of the population growth rate, even in populations that are only structured by age.

### Life history following a dynamic energy budget model

The method to integrate dynamic equations for life-history quantities, such as survival, simultaneously with the integration of the ODE that yields the value of Lotka's integral can be extended to apply the computational approach to more complicated models of individual life history. As an example I use here a simplified version of the DEB model that was originally developed by Kooijman (2000). In this model growth, reproduction and survival is dependent on individual body size and food availability in the environment. Compared to the original life-history model I have neglected all model parts that apply to starvation conditions as well as dynamics of energy reserves, because under conditions of constant food these parts do not apply or can be related directly to food availability. In the simplified model (see Table 2 for its definition) individuals are then characterized by three individual state variables (also referred to as *i*-state variables; Metz & Diekmann 1986): body size *V*, here expressed in terms of body volume, the hazard or mortality rate *h* of the individual and the amount *Q* of damage-inducing compounds (e.g. free radicals) that increase the individual's hazard rate. Growth in body size depends on size itself and on the scaled food intake rate *f*, which represents a measure of the food availability in the environment. The amount of damage-inducing compound is assumed to increase proportional to the energy expenditure on growth and maintenance, while the hazard or mortality rate increases proportional to the density of damage-inducing compounds per unit body volume. Individuals are assumed to reproduce only when larger than a threshold size *V*_p_. The energy investment into reproduction is determined on the basis of a careful accounting of various energy-consuming processes, including energy needed to maintain the individual's state of maturity, and therefore is a complicated function of body size and food availability. Table 2 lists the model equations and parameters with default values, as presented by Klanjscek *et al.* (2006). For a detailed presentation of the DEB model see Kooijman (2000), Nisbet *et al.* (2000), Muller & Nisbet (2000) and Klanjscek *et al.* (2006).

**Table 2:** Model variables, parameters with default value and interpretation, and equations describing the dynamic energy budget model, adapted from Klanjscek *et al.* (2006)

Variable	Dimension		Description
*V*	Length^3^		Volume of the structure compartment
*Q*	Mass		Mass of damage-inducing compound
*h*	Probability/time		Hazard or mortality rate: probability of death per unit time
**Parameter**	**Dimension**	**Value**	**Description**
κ	–	0.8	Energy partitioning coefficient
κ_*R*_	–	0.001	Fraction of reproduction energy realizedin a newborn
ν	Length/time	0.075 m.year^−1^	Energy conductance
*m*	Time^−1^	0.58 year^−1^	Maintenance rate coefficient: cost ofmaintenance relative to cost of growth
*g*	–	1.286*	Energy investment ratio: cost of growthrelative to maximum available energyfor growth
*V*_b_	Length^3^	10^−9^ m^3^	Structural volume at birth
*V*_p_	Length^3^	1.73 × 10^−6^ m^3^	Structural volume at maturation
[*E*_*m*_]	Energy/length^3^	0.7 *x*† J m^−3^	Maximum energy reserve density
*h*_*a*_	Length/mass/time	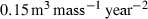	Ageing acceleration – rate of increase of thehazard rate
*f*	–	Varied	Energy intake scaled to maximumenergy intake

**Life-history equations and derived parameters**		
Rate of change in structural volume:	*G*(*f*,*V*) = (*fνV*^2/3^ − *mgV*)/(*f* + *g*)	(21)
Energy flux to reproductive processes:	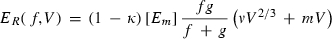	(22)
Energy flux required to maintain maturity:	*E*_*M*_ = (1 − κ)[*E*_*m*_]*mgV*_p_	(23)
Energetic costs of one newborn:	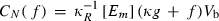	(24)
Reproduction rate:	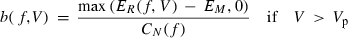	(25)
Production rate of damage-inducing compounds:	*D*(*f*,*V*) = *g*[*E*_*m*_](*G*(*f*,*V*) + *mV*)	(26)
Rate of increase of the hazard rate:	*H*(*Q*,*V*) = *h*_*a*_*Q*/*V*	(27)

* Value misprinted in original article (T. Klanjscek, personal communication).

† The factor *x* converts a chosen measure for energy into Joules, which cancels out after parameterization (Fujiwara *et al.* 2004; Klanjscek *et al.* 2006).

In the DEB model the age at first reproduction is not a constant parameter but depends on the environment, in particular the food density, that the individual experiences. In addition, both adult fecundity and the stable age distribution only depend indirectly on age through their dependence on body size and individual hazard rate, respectively. Nonetheless, the constant food conditions that are assumed ensure unique relationships between age and any of the three *i*-state variables, which only depend on the individual's state at birth and food conditions it experiences throughout life (Kirkilionis *et al.* 2001; Diekmann *et al.* 2003). These unique relationships between age and *i*-state allow for computation of the integral in Lotka's equation by integrating the following system of ODEs: (10)
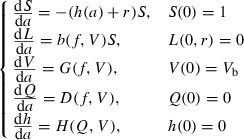
The ODEs for the variables *V*, *Q* and *h* in this system as well as their initial values at age 0 are determined by the life-history model (Klanjscek *et al.* 2006), while the first two ODEs are analogous to the system of equations for the Medfly example (eqn 9). Integrating this system to the maximum age *A*_m_ results in the single quantity of interest: (11)
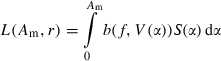


**Figure 1:**
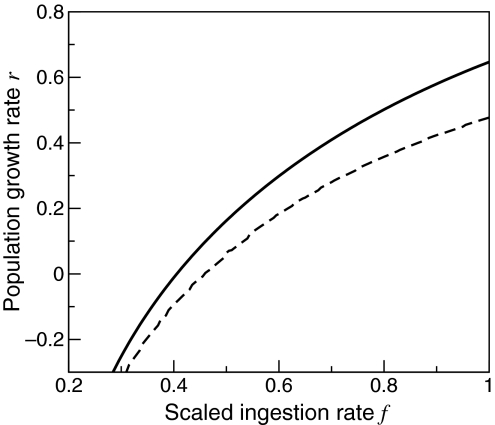
Population growth rate 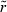
as a function of scaled food intake rate *f* for the dynamic energy budget model with continuous (solid line) and pulsed reproduction (dashed line).

As before the population growth rate 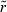
is the value of *r* that makes the integral *L*(*A*_m_,*r*) equal to 1 (eqn 5), which has to be obtained by an iterative solution method. The stable age distribution follows directly from integrating the system of ODEs in eqn 10 using 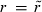, while reproductive value and sensitivities of 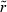
to changes in parameters can be computed in the same way as explained above (eqns 7 and 8, respectively). Figure 1 shows the population growth rate 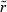
for the DEB model (Table 2) at different values of the food availability *f*. These results were calculated following the procedure outlined above with the matlab code provided in Appendix S5. Clearly, increasing food density translates into larger population growth rates, while the population growth becomes negative below *f* ≈ 0.4.

### More complex life-history models

The previous sections illustrate how the integration approach can be applied to the most common types of life-history models that are structured by either age or size. However, the method is sufficiently flexible that it can also be applied to a range of more complex cases. In this section I discuss some of these more complex applications without presenting all their mathematical details. Relevant equations are presented in Appendix 2.

#### Pulsed reproduction

Although the preceding presentation focuses on populations with continuous reproduction, the method can also be applied in case reproduction occurs as discrete events during life history. Matrix models may seem a more natural choice for demographic analysis of such birth-pulse populations, but deriving the elements of the projection matrix can be complicated if life-history development is continuous in time. For example, consider the DEB model as specified by the system of ODEs in eqn 10, but assume that reproduction only occurs at regularly spaced ages *A*_*i*_ = *i*Δ. Furthermore, assume that the number of offspring produced at age *A*_*i*_ is the product of the cumulative energy investment into reproduction between age *A*_*i*−1_ and age *A*_*i*_ and the probability to survive until age *A*_*i*_. Modelling a birth-pulse population that follows such DEB dynamics with a matrix model would require, among others, the specification of matrix elements that represent the survival probability from age *A*_*i*−1_ to age *A*_*i*_. Explicit expressions for these matrix elements are, however, lacking, because the DEB model only specifies survival in an indirect way by means of an ODE for the individual hazard rate. In contrast, computation of the population growth rate *r* is relatively straightforward with the integration approach using a system of ODEs very similar to eqn 10. As before, the growth rate 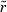
is the (unique) root of the equation *L*(*A*_m_,*r*) = 1. In this equation *L*(*A*_m_,*r*) again represents the expected, cumulative number of offspring that an individual produces during its life time, whereby the offspring production at each age *a* is discounted by the factor exp(−*ra*). Because of the pulsed reproduction, however, *L*(*A*_m_,*r*) has to be computed by summing up the offspring productions at the regularly spaced ages *A*_*i*_ = *i*Δ as opposed to its computation by integrating an ODE in case of continuous reproduction (see Appendix 2 for details).

Figure 1 shows the population growth rate 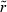
for the DEB model with pulsed reproduction at different values of the food availability *f* and compares it with the growth rate in case of continuous reproduction. The results with pulsed reproduction were calculated following the procedure outlined in detail in Appendix 2 with the matlab code provided in Appendix S6. Clearly, pulsed reproduction leads to a significantly lower population growth rate than continuous reproduction. As a consequence, pulsed reproduction requires a roughly 10% higher food availability to realize zero population growth. Pulsed reproduction affects population growth in two ways: when individuals die some reproductive investment goes to waste that does contribute to population growth in case of continuous reproduction. In addition, pulsed reproduction causes a delay between the moment of reproductive investment and the subsequent production of offspring, whereas offspring production follows reproductive investment immediately in case of continuous reproduction. These two factors are the only differences between the DEB models with pulsed and continuous reproduction and are hence jointly responsible for the lower population growth rate in case of pulsed reproduction.

#### Multiple types of individuals

A basic assumption in the models discussed above is that there is no variability among newborn individuals, for example, in the DEB model all individuals are born with the same body size *V*_b_. As a consequence, individuals in the exponentially growing population that are born at the same time will remain identical to each other throughout their entire life. The computational approach can, however, also be applied when there is variation among newborn individuals in their size at birth or in their life-history parameters. Such variation may induce that similarly aged individuals diverge during their life history. Consider that individuals can be born with one of a range of *N* potential sizes at birth. Adult individuals will hence potentially produce *N* different types of offspring. Let *L*_*ij*_(*A*_m_,*r*) represent the cumulative number of offspring with size at birth *i* produced by an individual that itself had size at birth *j*, in which the offspring produced at each age is discounted by the factor exp(−*ra*). Apart from the discrimination on the basis of the birth size of both mother and offspring, the elements *L*_*ij*_(*A*_m_,*r*) are analogous to the integral *L*(*A*_m_,*r*) of the basic DEB model (eqn 11). Together these elements constitute a matrix: (12)
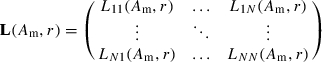


To compute the matrix entries a system of ODEs has to be integrated numerically for each of the *N* different sizes at birth describing the life-history development of an individual born with that particular size. These systems of ODEs are analogous to the systems of ODEs in eqns 9 and 10, but for the fact that they should keep track of how many offspring are produced of each possible type (see Appendix 2 for details). The population growth rate 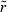
now corresponds to the largest root of the equation: (13)
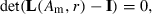


as can be inferred from Diekmann *et al.* (2003), who discuss the same generalization of a single to multiple states at birth for the computation of steady-states in density-dependent physiologically structured population models. Equation 13 is a generalized version of eqn 5 that allows the population to consist of finitely many different types of individuals. As before, it is an equation in the single unknown variable *r*, which can be computed using iterative root-finding methods, such as the secant or Newton-Raphson method (Press *et al.* 1988). However, unlike the quantity *L*(*A*_m_,*r*) in the DEB example the determinant det(**L**(*A*_m_,*r*) − **I**) is not necessarily a monotonously decreasing function of *r* and may have multiple roots *r*, of which the largest, dominant root is the required population growth rate.

Once the population growth rate 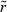
has been obtained the right eigenvector of the matrix 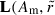) corresponding to the eigenvalue 1 represents the distribution of newborn individuals over the different states at birth, while its left eigenvector characterizes the reproductive value of newborn individuals with different states at birth (see Appendix 2 for more details). Finally, analogous to the expression in eqn 8 the sensitivity of the population growth rate 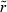
with respect to a particular parameter *p* can be computed as: (14)
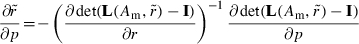
while elasticities follow from them as before. The differentials occurring in the above expression can in general not be obtained analytically and hence have to be approximated numerically (refer to the discussion of eqn 8). In practical applications Jacobi's formula for the derivative of a determinant, ∂det(***A***)/∂*x* = tr(adj(***A***)∂***A***/∂*x*), can be exploited for computing these derivatives, in which adj(***A***) is the adjugate of the matrix **A**.

#### Periodic environments

The computational approach can deal with populations, living in an environment that varies periodically in time, in the same way as populations consisting of different types of individuals are handled. Let the variable *E* denote the condition of the environment. In periodic environments this condition is a function of time with periodicity *T*, as expressed by *E*(*t* + *T*) = *E*(*t*) for all *t*. In the long run the population will grow exponentially, but with a periodic modulation that is enforced by the cyclic environmental conditions. As a consequence, the state of an individual at age *a* is not constant, but varies in time as well. In other words, individuals follow different life histories depending on the timing of their birth within the environmental cycle. The phase in the environmental cycle, at which it is born, can hence be considered as representing the individual's state at birth. Individuals are born continuously throughout the environmental cycle and the phase at which they are born hence varies continuously as well. Yet, the method can only handle finitely many different states at birth, which requires a discretization of the phase in the environmental cycle. To handle the periodic environment I will assume that the environmental cycle period *T* is discretized into *N* equal time intervals of length Δ and denote the phases of these discrete-time points within the cycle by phi _*i*_, such that phi _*i*_ = (*i* − 1)Δ = (*i* − 1)*T*/*N* (*i* = 1,…,*N*). Individuals can only be born at these discrete-time points, such that the continuous reproduction process is approximated by a set of finely spaced reproduction pulses at the phases phi _*i*_. For all *N* phases phi _*i*_ a system of ODEs has to be solved, which describes the life history of an individual born at that particular phase in the environmental cycle and which keeps track of the number of offspring produced by each individual at the different phases in the environmental cycle.

I illustrate this case of periodic environments with a variant of the Medfly model, in which juvenile medflies are periodically exposed to a very high mortality rate that decays exponentially within a short time period. Such a scenario could, for example, reflect a periodic treatment of the population with an insecticide that affects all juvenile individuals equally, irrespective of their age. Hence, the environmental condition *E*(*t*) in this example represents the additional juvenile mortality rate at time *t*, defined as 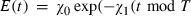)). Mathematical and computational details for this model are given in Appendix 2.

**Figure 2:**
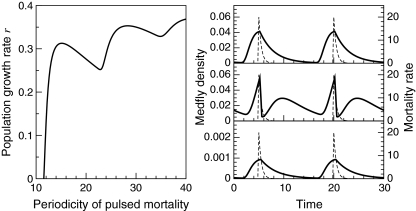
Left: Population growth rate of the Medfly life-history model when juveniles are exposed to strong, periodic pulses of mortality as a function of the period between these mortality pulses. Right: Pulses of mortality (thin dashed line), changes in population birth rate (top) and changes in relative density of juvenile (middle) and adult (bottom) medflies (all thick solid lines) in the exponentially growing population during the cycles of additional juvenile mortality. Period of mortality pulses: 15 days. All results were obtained assuming a peak juvenile mortality of khgr _0_ = 20 day^−1^, which decays rapidly over time with a time constant of khgr _1_ = 2 day^−1^, and a discretization of the phase in the environmental cycle into intervals with length Δ = 0.2 (halving or doubling the latter value does not noticeably change the results).

Figure 2 (left panel) shows the population growth rate of the Medfly population as a function of the period between two pulses of high juvenile mortality. The results were calculated following the procedure outlined above (see also Appendix 2) with the matlab code provided in Appendix S7. Obviously, the population growth rate increases with longer periods between mortality pulses. However, the relationship is not monotonous and exhibits some distinct peaks at particular periods. For example, a period of 15 days between consecutive mortality peaks yields a significantly higher population growth rate than periods that are somewhat longer or shorter. The peaks and troughs in the relationship between population growth rate and the mortality periodicity come about because of an interplay between the periodicity and the juvenile period of the Medfly. The right panels of Fig. 2 show for the peak population growth rate at a periodicity of 15 days the changes in relative composition in the exponentially growing population during the period between mortality pulses. Recruitment to the adult stage is high around the time of a pulse in juvenile mortality. These newly matured adults immediately cause a strong increase in the population reproduction rate and the density of juveniles. The first wave of this offspring is exposed to the pulse of high mortality and hence dies rapidly, killing roughly 90% of them. However, the offspring that the newly matured adults produce slightly later escape the high mortality levels and are born sufficiently early that they make it to maturation (11 days later) before the next pulse of juvenile mortality occurs. Had the period between the pulses been slightly shorter, they would have been hit by the subsequent mortality pulse before maturation, had the period been slightly longer most of their offspring would have been produced already and would hence have suffered from the subsequent mortality pulse. The peak in population growth rate at a periodicity of 15 days hence results because a complete juvenile period fits exactly between the moment that mortality levels have dropped sufficiently after a pulse and before the next mortality pulse occurs. In the long run the exponentially growing population converges to this timing of maturation and reproduction as it yields the highest population growth rate.

## Discussion

The approach presented in this paper complements the development of matrix (Caswell 2001) and integral projection models (Easterling *et al.* 2000; Ellner & Rees 2006) for discrete-time dynamics by providing a method for demographic analyses of populations with continuous reproduction and development – so-called birth-flow populations. Previously, growth and demography of birth-flow populations have been studied using age- or stage-classified matrix models, which require an assumption about the projection and age-classification interval and relationships to express the matrix elements in terms of the continuous life-history functions (Caswell 2001). The Medfly example illustrates that the choice of the projection interval may significantly influence the estimate of the population growth rate and that taking a finer age classification does not necessarily provide a better estimate. Consequently, even in a relatively simple case, in which the individual life history is described in terms of an age-dependent survival and reproduction rate, the integration approach yields a more reliable estimate of the population growth rate.

Further complications arise when dynamics of a birth-flow population are represented with a stage-classified matrix model and stage transitions depend on a dynamic model of individual energetics (Klok & De Roos 1996; Klok *et al.* 1997; Klanjscek *et al.* 2006). The study by Klanjscek *et al.* (2006) provides an example, in which a stage-classified matrix model based on the DEB model in Table 2 was formulated to compute the population growth rate. To derive the matrix elements these authors make a number of simplifying assumptions: for example, the population is classified in only a juvenile and adult stage, reproduction is assumed to occur pulsed in time and individual fecundity is an averaged value of the reproductive investment over a time interval. Furthermore, the matrix elements depend on the stable age distribution within the juvenile and adult stage. Expressions for this stable age distribution are difficult to derive, since the death rate itself is a dynamic variable (eqn 10). The stable age distribution moreover depends on the value of the population growth rate, such that the formulation of the matrix and the computation of its eigenvalue have to be iterated until the estimate of the latter converges. In contrast, the integration approach allows for the computation of the population growth rate of the DEB model without making additional assumptions and without deriving an explicit expression for the stable age distribution.

For life-history models with continuous reproduction or development that are couched in terms of a system of ODEs, the integration approach thus appears to be a more straightforward and more reliable method for population growth rate analysis than an approach using matrix models. In the form presented here, however, the method only applies to demographic analysis of exponentially growing populations in environments that are either constant or fluctuate periodically in time. It does not allow for demographic analysis of populations that grow unboundedly in a stochastically fluctuating environment, nor does it allow for the analysis of transient dynamics. For matrix models of populations in stochastic environments techniques have been developed to compute the population growth rate (Tuljapurkar 1990) and to determine its sensitivity with respect to changes in demographic parameters (Haridas & Tuljapurkar 2005). Similarly, for discrete-time population models methods exist to compute the sensitivity analysis of transient dynamics (Caswell 2007). It is as of yet unclear whether variants of the methods presented in this paper can be developed to carry out these analysis for continuous-time, structured population models. Transient dynamics of such models can be studied, but require the formulation of a model in terms of partial differential equations (Metz & Diekmann 1986; De Roos 1997).

In density-dependent environments elasticity analysis of discrete-time models has focused on the change in population size in response to changes in demographic parameters (Grant & Benton 2003; Benton *et al.* 2004). Such analyses are also possible for density-dependent, physiologically structured population models in continuous time, as long as the population is at equilibrium. Diekmann *et al.* (2003, see also Kirkilionis *et al.* 2001; Claessen & De Roos 2003) derived methods to compute the steady-state of such models that are similar to the computational approach presented in this paper. These methods hence allow for assessing the change in equilibrium abundance in response to changes in life-history parameters. The computational approach presented here differs from the methods developed by Diekmann *et al.* (2003) in that feedback of the population on its environment and hence density dependence is ignored. The lack of density dependence results in exponential population growth and allows for an elasticity analysis of the rate of this exponential increase.

The integration approach developed in this paper is purely a numerical one and does not provide analytical expressions for the population growth rate. Analytical studies of the population growth rate in continuous-time models have been carried out in specific cases (e.g. Kooijman & Metz 1984; Takada & Caswell 1997), but are not applicable generally. Similarly, for matrix models analytical investigations of the population growth rate are possible (Caswell 2001), but the majority of studies deal with numerical calculations of the population growth rate and its associated statistics. I hence argue that the method presented in this paper is not essentially different from the methods for demographic analysis of matrix models. The method may seem complicated, but computational methods to solve for the eigenvalues and especially the eigenvectors of a matrix model are not very straightforward either. A major difference is that the latter methods are standard part of software packages like matlab, while for explanation and implementation of the computational method presented in this paper we have to start from scratch.

## Appendix 1

### Analytical calculations for the Medfly example

Given the exponentially increasing mortality rate with age (see Table 1), Medfly survival follows a Gompertz survival function (Carey 1993, p. 159):

Hence, the integral in Lotka's integral equation takes the form:

Changing integration variables by defining *y* = exp(mgr _1_(*a* − *A*_*j*_)) the integral can be expressed as:

In which *q*, *s* and *z* are defined as:

Using maple the integral can now be solved and written in terms of a Gamma function:

Substituting the expressions for *q*, *s* and *z* back into the right-hand side of this equation, leads to the following form of Lotka's integral equation for the Medfly life-history model:

I numerically solved this last equation for the population growth rate *r* after substituting the parameters values listed in Table 1. Furthermore, again using maple I symbolically calculated derivatives of the left-hand side of the equation with respect to *r*, bgr _0_, bgr _1_, *A*_*j*_, mgr _0_ and mgr _1_. From these derivatives the sensitivity of the population growth rate with respect to the five parameters was computed on the basis of eqn 8 after substituting the parameters values listed in Table 1. The results thus obtained were used for comparison with the results obtained by solving eqn 5 after integration of the system of ODEs in eqn 9.

I also constructed age-classified matrix models from the explicit expressions for *F*(*a*) and *b*(*a*) using the projection matrix:

All matrix elements *m*_*ij*_ equal 0, except those in the subdiagonal, representing survival, and those on the first row, representing reproduction. The subdiagonal elements were computed using the equation *m*_*i*+1,*i*_ = (*F*(*i*Δ) + *F*((*i* + 1)Δ))/(*F*((*i* − 1)Δ) + *F*(*i*Δ)) (Caswell 2001, eqn 2.24), while the elements in the first row were computed using *m*_1*i*_ = *F*(Δ/2)(*b*(*i*Δ) + *m*_*i*+1,*i*_*b*((*i* + 1)Δ))/2 (Caswell 2001, eqn 2.34). I varied the discretization interval Δ between 0.5, 1.0, 2.0 and 4.0 days. The dimension of the matrix was equal to *N* = 50/Δ, as 50 days was approximately the maximum age in the integration approach. The population growth rate was computed using matlab as *r* = log(lgr)/Δ with lgr the dominant eigenvalue of the matrix **M**.

## Appendix 2

### More complex life histories: mathematical details

#### Pulsed reproduction

In the DEB model with pulsed reproduction let *B*(*a*) denote the cumulative investment in offspring by an individual that has survived up to age *a*. Mathematically, *B*(*a*) equals the integral of *b*(*f*,*V*) from age 0 to age *a* (see Table 2) and can be computed by numerical integration of an ODE, analogous to the computation of *L*(*a*,*r*) in case of continuous reproduction. The entire life history of the individuals is determined by the following system of equations:

This system of ODEs does not include an ODE for *L*(*a*,*r*), as *L*(*A*_m_,*r*) is now computed as ). In this sum the difference *B*(*A*_*i*_) − *B*(*A*_*i*−1_) represents the number of offspring that is produced at age *A*_*i*_ on the basis of the reproductive allocation between *A*_*i*−1_ and age *A*_*i*_. However, only individuals that survive until age *A*_*i*_ can reproduce, which explains the multiplication with *S*(*A*_*i*_). *L*(*A*_m_,*r*) has to be computed by integration of the life-history ODEs in a stepwise manner from age *A*_*i*−1_ to age *A*_*i*_ and summation of the appropriate contributions (*B*(*A*_*i*_) − *B*(*A*_*i*−1_))*S*(*A*_*i*_). As before, the population growth rate *r* is determined by the equation *L*(*A*_m_,*r*) = 1.

#### Multiple types of individuals

I will discuss technical details regarding how to apply the integration approach to a case with multiple types of individuals using a variant of the DEB model, in which individuals are either born with size 0.7×*V*_b_ or alternatively with size 1.3×*V*_b_. These will be referred to as ‘type 1’ individuals and ‘type 2’ individuals, respectively. Furthermore, both types of individuals are assumed to spend 2/3 of their reproductive investment on producing offspring with their own birth size and 1/3 of their effort into offspring with the alternative type. matlab code for this particular example model is provided in Appendix S8.

Because their states at birth differ individuals born with a small or a large size both follow their own, unique life history, even though they experience the same constant food conditions. The life history of individuals of type 1 is determined by six variables: *S*_1_(*a*), *L*_11_(*a*,*r*), *L*_21_(*a*,*r*), *V*_1_(*a*), *Q*_1_(*a*) and *h*_1_(*a*), which follow dynamics similar to the system of ODEs in eqn 10 except that *V*_1_(0) = 0.7×*V*_b_:

(15)

In these equations *L*_11_(*a*,*r*) and *L*_21_(*a*,*r*) represent the cumulative number of small and large offspring, respectively, produced by an individual that itself had a small size at birth and weighted at every age by exp(−*ra*). The functions *b*_11_(*f*,*V*_1_) and *b*_21_(*f*,*V*_1_) represent the *per capita* rate at which the individual produces these two types of offspring and are defined as

respectively [compare the corresponding expression for *b*(*f*,*V*) in Table 2]. The division by 0.7×*V*_b_ and 1.3×*V*_b_ accounts for the fact that with the same amount of investment more individuals with a small size at birth can be produced. A comparable set of quantities that follow the same dynamics as specified above describe the life history of the individuals that are born with a large size at birth. The only difference is that for these individuals *V*_2_(0) = 1.3×*V*_b_.

After integrating the systems of ODEs for both types of individuals the following matrix:

is constructed, which allows computation of the population growth rate as the root of the condition det(**L**(*A*_m_,*r*) − **I**) = 0. Denote the right and left eigenvector of the matrix ) as **u** and **v**, respectively, such that and . If **u** is normalized such that its elements sum to unity, i.e. **1**^T^**u** = 1, this right eigenvector represents the distribution of the stable birth flow in the exponentially growing population over the various states at birth. In the current example the elements *u*_1_ and *u*_2_ of the right eigenvector, representing the fraction of all individuals born at a specific time with a small and large size at birth, respectively, are given by:

These expressions can be derived from the equation , assuming that *u*_1_ + *u*_2_ = 1. Given these fractions the stable age distribution of the population is given by:

in which *S*_1_(*a*) and *S*_2_(*a*) are obtained as results of the integration of the system of ODEs. The expression makes clear that individuals with age *a* are a mixture of individuals that were born with a small and large size at birth, respectively, and therefore differ in body size at this age as well.

The elements *v*_1_ and *v*_2_ of the left eigenvector **v** of the matrix ) are given by:

as can be deduced from the equation , assuming *v*_1_ + *v*_2_ = 1. With this latter normalization each component *v*_*j*_ can be interpreted as follows: suppose that at time *t* = 0 every possible state at birth is represented in the initial population by a single newborn individual. In the long run a fraction *v*_*j*_ of the total population will be descended from the newborn individual with state at birth *j*. The particular normalization of the left eigenvector **v** determines which type of newborn individual has the reference reproductive value of 1. More commonly, the left eigenvector is normalized such that **v**^T^**u** = 1 (Caswell 2001), which assigns this unit reproductive value to the ‘average’ newborn individual in the exponentially growing population. As *v*_1_ and *v*_2_ represent reproductive values at age 0, they are more appropriately denoted as *v*_1_(0) and *v*_2_(0), respectively. At a later age the reproductive value *v*_*j*_(*a*) of an individual with state at birth *j* can be calculated as the exponentially discounted, expected number of different types of offspring that the individual will still produce during its remaining life time. The different types of offspring have to be weighted, however, by the contribution these offspring make themselves to future population growth (compare eqn 7):

(16)

Notice that *v*_*j*_(0) = ∑_*i*_*v*_*i*_(0)*L*_*ij*_(*A*_m_,*r*) because **v** is the left eigenvector of the matrix ) and that *b*_*ij*_(*a*) represents the rate at which an individual of age *a*, which itself was born with state *j*, produces offspring with state at birth *i*. The expressions above indicate that the reproductive value can be computed from the quantities *S*_*j*_(*a*) and ) after integrating the appropriate set of life-history ODEs (cf. eqn 15) for the individual with state at birth *j* up to age *a*.

#### Periodic environments

In the variant of the Medfly example model with high, periodic pulses of juvenile mortality the environmental condition *E*(*t*), representing the additional juvenile mortality rate at time *t*, is defined as . Let *S*_*i*_(*a*) denote the stable age distribution at age *a* of individuals born at phase phi _*i*_ = (*i* − 1)Δ = (*i* − 1)*T*/*N* (*i* = 1,…,*N*). Furthermore, let *L*_*i*_(*a*,*r*) denote the cumulative offspring production, weighted with the factor exp(−*ra*) at every age, produced by this individual up to age *a*. The dynamics of *S*_*i*_(*a*) and *L*_*i*_(*a*,*r*) are described by the following system of ODEs:

(17)

This system is analogous to the system of equations for the Medfly example in constant environments (eqn 9) except that the death rate is increased with *E*(*a* + phi _*i*_), which reflects that the death rate at age *a* is a function of the phase in the environmental cycle at which individuals reach this particular age. For all *N* phases phi _*i*_ an analogous system of ODEs has to be solved, which only differ in their value of *E*(*a* + phi _*i*_), i.e. the level of mortality they experience at a particular age *a*. Handling the periodic environment in case of the Medfly example therefore requires the simultaneous integration of 2*N* differential equations as opposed to the two ODEs that are needed in constant environments.

Let *L*_*ij*_(*a*,*r*) now denote the number of offspring, weighted by exp(−*ra*) at every age, produced at phase phi _*i*_ in the environmental cycle by an individual that was itself born at phase phi _*j*_. To compute these quantities *L*_*ij*_(*a*,*r*) the numerical integration of the system of 2*N* ODEs has to be carried out in a stepwise manner from *A*_*l*−1_ = (*l* − 1)Δ to *A*_*l*_ = *l*Δ(*l* = 1, 2, 3,…). At age *A*_*l*_ = *l*Δ an individual that is born at phase phi _*j*_ in the environmental cycle produces a number of offspring equal to the cumulative number of offspring produced within the just elapsed interval Δ. Given its own phase at birth and its age this offspring is produced at phase phi _*i*_ with . At *A*_*l*_ = *l*Δ the following operation hence has to be carried out for all *j* =1, …, *N*:

(18)

The integration of the ODEs (eqn 17) and the recurring summations (eqn 18) have to be carried out until reaching *a* = *A*_m_, as was also the case for the constant environment model. The final results of the integration are the elements *L*_*ij*_(*A*_m_,*r*) of the matrix **L**(*A*_m_,*r*) (eqn 12). All computations with this matrix can subsequently be carried out as described in detail in the section on multiple types of individuals. In particular, the population growth rate can be obtained from solving eqn 13. Furthermore, the right eigenvector belonging to the unit eigenvalue of the matrix ) constructed with the correct value of the population growth rate represents the relative densities of individuals with different states at birth. In periodic environments this eigenvector is hence proportional to the rate at which individuals in the exponentially growing population are born at the different phases phi _*i*_ within the environmental cycle.

## SUPPLEMENTARY MATERIAL

The following supplementary material is available for this article:

Appendix S1 MATLAB code to compute the population growth rate.

Appendix S2 MATLAB code to compute the sensitivity of the population growth rate to all model parameters.

Appendix S3 MATLAB code to compute the entire life history as a function of individual age for computation of the stable age distribution and the reproductive value as a function of age.

Appendix S4 Problem-specific MATLAB code for the Medfly example.

Appendix S5 Problem-specific MATLAB code for the dynamic energy budget example with the population made up by a single type of individual (a single state at birth).

Appendix S6 Problem-specific MATLAB code for the dynamic energy budget example with the population made up by a single type of individual (a single state at birth) and pulsed reproduction.

Appendix S7 Problem-specific MATLAB code for the Medfly example with a periodically fluctuating juvenile mortality.

Appendix S8 Problem-specific MATLAB code for the dynamic energy budget example with the population consisting of two different types of individuals (two states at birth). The code in Appendix S1–S3 is designed to be problem independent. For specific problems and parameter combinations the code might nonetheless fail to yield results and should therefore be considered a starting point for further development.

This material is available as part of the online article from:

http://www.blackwell-synergy.com/doi/full/10.1111/j.1461-0248.2007.01121.x
